# Autophagy and Mitophagy Promotion in a Rat Model of Endometriosis

**DOI:** 10.3390/ijms22105074

**Published:** 2021-05-11

**Authors:** Rosalba Siracusa, Ramona D’Amico, Daniela Impellizzeri, Marika Cordaro, Alessio Filippo Peritore, Enrico Gugliandolo, Rosalia Crupi, Angela Trovato Salinaro, Emanuela Raffone, Tiziana Genovese, Salvatore Cuzzocrea, Roberta Fusco, Rosanna Di Paola

**Affiliations:** 1 Department of Chemical, Biological, Pharmaceutical and Environmental Sciences, University of Messina, 98166 Messina, Italy; rsiracusa@unime.it (R.S.); rdamico@unime.it (R.D.); dimpellizzeri@unime.it (D.I.); aperitore@unime.it (A.F.P.); rfusco@unime.it (R.F.); dipaolar@unime.it (R.D.P.); 2 Department of Biomedical, Dental and Morphological and Functional Imaging, University of Messina, Via Consolare Valeria, 98125 Messina, Italy; cordarom@unime.it; 3 Department of Veterinary Sciences, University of Messina, 98168 Messina, Italy; egugliandolo@unime.it (E.G.); rcrupi@unime.it (R.C.); 4 Department of Biomedical and Biotechnological Sciences, University of Catania, 95124 Catania, Italy; trovato@unict.it; 5 Multi-Specialist Istitute Rizzo, Torregrotta, 98043 Messina, Italy; emanuelaraffone@virgilio.it

**Keywords:** autophagy, mitophagy, endometriosis

## Abstract

Endometriosis is a gynecological condition affecting patients in reproductive age. The aim of this paper was to assess the effects of the autophagy and mitophagy induction in a rat model of endometriosis. Endometriosis was induced by the injection of uterine fragments, and rapamycin (0. 5 mg/kg) was administered once per week. One week from the induction, rats were sacrificed, and laparotomy was performed to collect the endometriotic implants and to further process them for molecular analysis. Western blot analysis was conducted on explanted lesions to evaluate the autophagy pathway during the pathology. Elevated phospho-serine/threonine kinase (p-AKT) and mammalian target of rapamycin (mTOR) expressions were detected in vehicle-treated rats, while Beclin and microtubule-associated protein 1A/1B-light chain 3 II (LC3II) expressions were low. Additionally, samples collected from vehicle groups indicated low Bnip3, Ambra1, and Parkin expressions, demonstrating impaired autophagy and mitophagy. Rapamycin administration reduced p-AKT and mTOR expressions and increased Beclin and LC3II, Bnip3, Ambra1, and Parkin expressions, activating both mechanisms. We also evaluated the impact of the impaired autophagy and mitophagy pathways on apoptosis and angiogenesis. Rapamycin was administered by activating autophagy and mitophagy, which increased apoptosis (assessed by Western blot analysis of Bcl-2, Bax, and Cleaved-caspase 3) and reduced angiogenesis (assessed by immunohistochemical analysis of vascular endothelial grow factor (VEGF) and CD34) in the lesions. All of these mechanisms activated by the induction of the autophagy and mitophagy pathways led to the reduction in the lesions’ volume, area and diameter.

## 1. Introduction

Endometriosis is a painful, debilitating and chronic disease in which endometrium-like stromal and glandular cells grow outside of the uterus (including the pelvic peritoneum, ovaries and rectovaginal septum) [1,2]. It is an estrogen-dependent and inflammatory disease that affects 7–11% of women during their reproductive period and up to 40% of women receiving fertility treatments [3]. It is the third cause of hospitalization in the US [3]. According to Sampson’s hypothesis, the most accredited theory, during menses, shed endometrial tissues exit from the uterine cavity through the fallopian tubes and reach the peritoneal cavity by retrograde menstruation [4,5,6]. These shed endometrial tissues implant, survive and grow at ectopic places, transforming into endometriotic lesions [5,7].

Other theories have proposed that endometriosis originates from extrauterine cells that abnormally transdifferentiate or transform into endometrial cells. The coelomic metaplasia theory postulates that endometriosis originates from the metaplasia of specialized cells that are present in the mesothelial lining of the visceral and abdominal peritoneum. According to this theory, residual embryonic cells of the Wolffian or Mullerian ducts persist and develop into endometriotic lesions that respond to estrogen [8]. Furthermore, recent theories suggest coelomic metaplasia to be the origin of an adolescent variant of a severe and progressive form of endometriosis [9]. In normal conditions, upon detachment from the extracellular matrix, epithelial cells undergo anoikis [10]. This mechanism of programmed cell death is carefully regulated to maintain cellular homeostasis [11,12]. Accordingly, the development of endometriosis is related with the migration and survival of endometrial stromal cells outside the uterus. During endometriosis the anoikis response is altered by autophagy [13]. In particular, altered autophagic flux induced changes in cellular survival [11,12]. Autophagy is a complex pathway that starts with double-membrane vesicles formation, known as autophagosomes, which surround cytoplasmic components. Lysosomes fuse with autophagosomes to recycle and degrade their cargo composed of lipids, oxidized proteins and damaged organelles. Depending on the way the protein is delivered to the lysosome, four basic types of autophagy can be distinguished: macroautophagy, selective autophagy, chaperone-mediated autophagy and microautophagy [14]. Macroautophagy involves the formation of autophagosomes and is controlled by specific autophagy-related genes. The steps in macroautophagy are initiation, phagophore elongation, autophagosome maturation, autophagosome fusion with the lysosome and proteolytic degradation of the contents. Selective autophagy is macroautophagy of a specific cellular component. In chaperone-mediated autophagy, the substrate is bound to a heat shock protein 70 chaperone before it is delivered to the lysosome. The least characterized type of autophagy is microautophagy, which is the degradation of very small molecules without participation of an autophagosome. This work focuses on mitophagy (selective autophagy of abnormal and damaged mitochondria), in which the main participating protein is PINK1 (phosphatase and tensin homolog-induced putative kinase 1).

Several papers evidence the role of autophagy in the progression and development of endometriosis [15,16,17,18,19,20,21]. In a mouse model of surgically induced endometriosis, ectopic lesions revealed increased levels of autophagy-related protein 9A (ATG9A), an autophagic protein involved in vesicle formation [22,23]. Analysis conducted on human endometriomas lesions (ovarian endometriosis) revealed a reduction in microtubule-associated protein 1A/1B-light chain 3 II (LC3-II) expression when compared to control endometrial tissue [24]. Reduced Beclin-1 expression was detected in samples from endometriosis patients when compared to cultured stromal cells [25,26]. Additionally, it has been demonstrated by in vivo experiments that during endometriosis, the inhibition of autophagy by increasing extracellular signal-regulated kinase (ERK), mammalian target of rapamycin (mTOR) and serine/threonine kinase (AKT)/mTOR activity further downregulates apoptosis [27]. Endometriotic lesions from patients revealed decreased expressions of pro-apoptotic factors and increased expressions of anti-apoptotic factors when compared with healthy women [17,28]. Impaired apoptosis further contributes to the survival of endometrial cells into the peritoneal cavity and establishment of endometriosis. Impaired apoptosis is often related with increased angiogenesis, which supports the growth and development of the lesions in mice [16].

In particular, it has been described that autophagy activators would inhibit the growth of endometriotic lesions, most possibly through the inhibition of the expression of VEGF in lesions, thereby inhibiting angiogenesis [16]. A key function of autophagy is the removal of aged or damaged organelles, such as mitochondria. Mitochondrial autophagy, or mitophagy, is the degradative mechanism for the elimination of damaged mitochondria [29]. In particular, the elimination of these organelles by autophagy requires two steps: activation of general autophagy and priming of injured mitochondria for selective autophagy recognition. Mitophagy priming is mediated by mitophagic receptors Bnip3 and Nix or the Pink1-Parkin signaling pathway. Several experimental papers described a mechanistic link between autophagy, mitochondrial turnover and cell survival and proliferation [18,19]. mTOR is a serine/threonine kinase and is a critical inhibitor of autophagy. It has been described as managing the autophagy regulation and the mitochondrial health under pathological and physiological conditions [30,31]. Rapamycin is a macrocyclic antibiotic produced by the bacterium *Streptomyces hygroscopicus*. It is commonly used as an immunosuppressant, antifungal and antitumor drug that blocks mTOR [32]. Nowadays, targeting the mTOR axis with the existing drugs is not acceptable for endometriosis management, given its ubiquitous role and the associated side effects. In this paper we aimed to evaluate the effects of the autophagy and mitophagy promotion in a rat model of endometriosis.

## 2. Results

### 2.1. Effect of Autophagy and Mitophagy Activation on Lesion Size in Endometriosis

**Figure 1:**
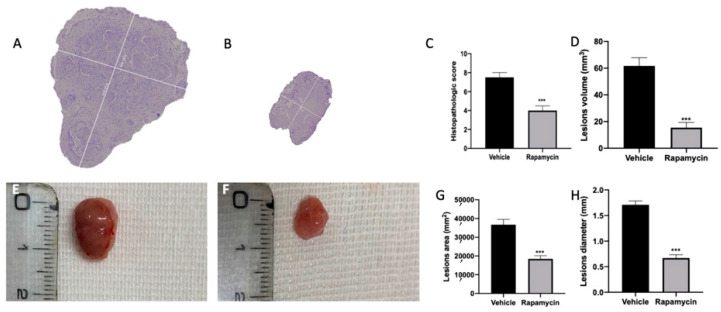
Mitophagy activation reduced endometriosis-induced lesion size. Histological analysis: vehicle (**A**), rapamycin (**B**), histopathological score (**C**), lesion volume (**D**). Macroscopic analysis: vehicle (**E**), rapamycin (**F**), cysts area (**G**), cysts diameter (**H**). For the analyses, *n* = 5 animals from each group were employed. A p value of less than 0.05 was considered significant. *** *p* < 0.001 vs. vehicle.

Seven days after endometriosis induction, animals from vehicle (Figure S1A) and rapamycin (Figure S1B) groups showed lesion development. No differences between the groups were shown (Figure S1C,D). At 14 days after endometriosis, rapamycin administration reduced the histopathological score of the lesions (Figure 1B,C (*p* < 0.0001)) as compared to the vehicle group (Figure 1A,C). Additionally, rapamycin significantly reduced lesion volume (Figure 1D (*p* < 0.0001)), area (Figure 1G (*p* < 0.0001)) and diameter (Figure 1H (*p* < 0.0001)) as evidenced by the macroscopic analysis (Figure 1F) as compared to the vehicle group (Figure 1E), while sham animals did not show any implants.

### 2.2. Autophagy Inhibition Induced by Endometriosis

**Figure 2:**
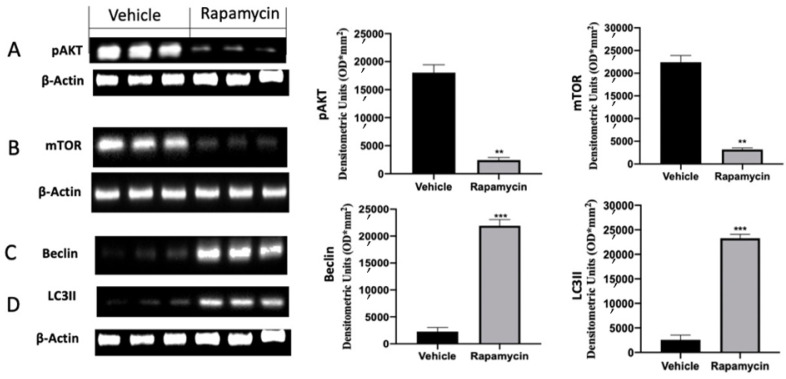
Evaluation of autophagy induction: Western blot analysis of phospho-serine/threonine kinase (p-AKT) (**A**), mammalian target of rapamycin (mTOR) (**B**), Beclin (**C**) and microtubule-associated protein 1A/1B-light chain 3 II (LC3II) (**D**) expression. For the analyses, *n* = 5 animals from each group were employed. A p value of less than 0.05 was considered significant. ** *p* < 0.01 vs. vehicle, *** *p* < 0.001 vs. vehicle.

Western blot analysis was conducted on explanted lesions to evaluate the autophagy pathway during the pathology. Samples collected from vehicle groups showed increased p-AKT and mTOR activation. Rapamycin administration induced a significant reduction in both proteins, inducing autophagy activation (Figure 2A (*p* = 0.0042) and Figure 2B (*p* = 0.0040)). Well in line with these results, vehicle groups revealed reduced Beclin and LC3II expressions, which were significantly increased after rapamycin administration (Figure 2C (*p* = 0.0003) and Figure 2D (*p* < 0.0001)).

### 2.3. Mitophagy Inhibition Induced by Endometriosis

**Figure 3:**
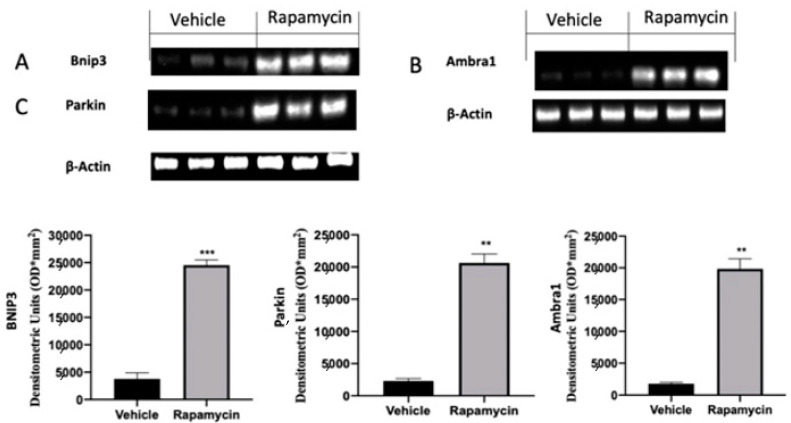
Evaluation of mitophagy induction: Western blot analysis of Bnip3 (**A**), Ambra1 (**B**) and Parkin (**C**) expression. For the analyses, *n* = 5 animals from each group were employed. A p value of less than 0.05 was considered significant. ** *p* < 0.01 vs. vehicle, *** *p* < 0.001 vs. vehicle.

In the next step of the paper we evaluated the role of the mitophagy mechanism during endometriosis. Vehicle groups showed impaired mitophagy mechanisms, as revealed by the Western blot analysis. Samples collected from vehicle groups showed low Bnip3 (Figure 3A), Ambra1 (Figure 3B) and Parkin (Figure 3C) expressions. The activation of the autophagy pathway by rapamycin in turn also activated mitophagy. Bnip3 (Figure 3A (*p* = 0.0002)), Ambra1 (Figure 3B (*p* = 0.0069)) and Parkin (Figure 3C (*p* = 0.0039)) levels were significantly increased in rapamycin administered rats.

### 2.4. Apoptosis Inhibition Induced by Endometriosis

**Figure 4:**
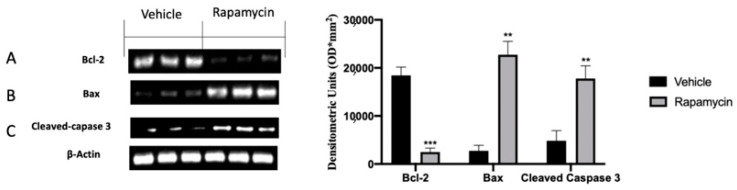
Evaluation of apoptosis induction: Western blot analysis of Bcl-2 (**A**), Bax (**B**) and Cleaved-caspase 3 (**C**) expression. For the analyses, *n* = 5 animals from each group were employed. A p value of less than 0.05 was considered significant. ** *p* < 0.01 vs. vehicle, *** *p* < 0.001 vs. vehicle.

One of the key molecular pathways inhibited during endometriosis is the apoptosis mechanism. According to this concept, the analysis of the endometriotic lesions collected from vehicle groups showed impaired apoptosis. In particular, we noted increased Bcl-2 expression in vehicle groups, and in turn lox Bax (Figure 4B) and Cleaved-caspase 3 levels (Figure 4C). Rapamycin administration significantly increased apoptosis in the lesions, as displayed by the reduced Bcl-2 levels (Figure 4A (*p* = 0.0009)) and increased Bax (Figure 4B (*p* = 0.0025)) and Cleaved-caspase 3 expressions (Figure 4C (*p* = 0.0033)). In particular, rapamycin reduced pro-apoptotic Bcl-2 expressions in a more significant way as compared to Bax and Cleaved-caspase 3.

### 2.5. Angiogenesis Induction Induced by Endometriosis

**Figure 5:**
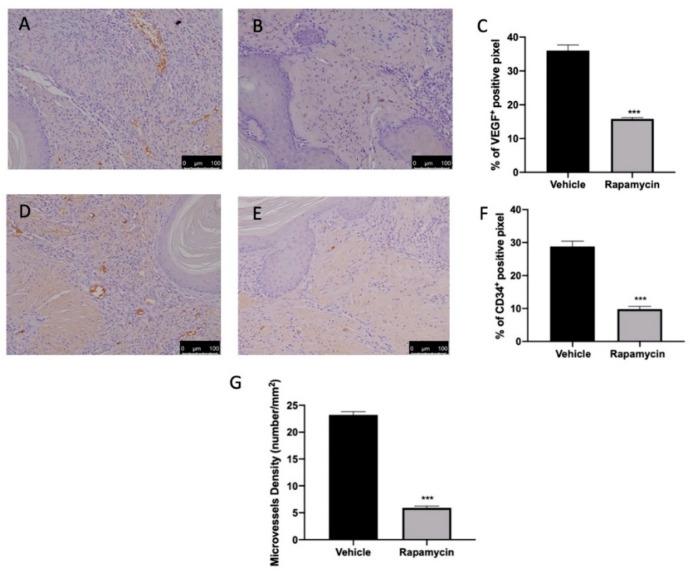
Evaluation of angiogenesis induction. Immunohistochemical analysis of VEGF expression: vehicle (**A**), rapamycin (**B**), graphical quantification of VEGF expression (**C**). Immunohistochemical analysis of CD34: vehicle (**D**), rapamycin (**E**), graphical quantification of CD34 expression (**F**). Microvessel density (**G**). For the analyses, *n* = 5 animals from each group were employed. A p value of less than 0.05 was considered significant. *** *p* < 0.001 vs. vehicle.

Ramapycin administration was also able to reduce angiogenesis in the lesions. Immunohistochemical analysis revealed elevated VEGF (Figure 5A,C) and CD34-positive (Figure 5D,F) expressions in tissues collected from vehicle groups. Ramapycin administration significantly reduced both markers of angiogenesis (Figure 5B,C (*p* < 0.0001) and Figure 5E,F (*p* < 0.0001)) and microvascular density in the lesions (Figure 5G (*p* < 0.0001)).

## 3. Discussion

In this study we demonstrated that endometriosis development required impaired autophagy and mitophagy. In addition, compromised autophagic influx was related to inhibited apoptosis and increased angiogenesis. Autophagy had a role in many physiological mechanisms, including elimination of misfolded or aggregated proteins, starvation, anti-aging, cell growth and innate immunity. Thus, dysregulation of autophagy has been found in multiple diseases, including cancer, muscular, cardiovascular and neurodegenerative disorders [33,34,35,36]. Recently, many papers focused on the relationship between autophagy and endometriosis. This degradative and selective process is mediated by the phosphatidylinositol 3-kinase (PI3K)/Akt/mTOR pathway [37].

The current study investigated the underlying effects on mitochondria during mTOR signaling suppression and identified mitochondrial parameters that could be targeted therapeutically.

Our results showed that the induction of the autophagy and mitophagy pathways lead to the reduction in the lesions’ volume, area and diameter.

Akt and mTOR up-regulation in endometriotic tissues showed an impaired autophagic response in this disease, which resulted in a promoted survival of dethatched endometriotic cells. Endometriotic lesions also showed reduced Beclin-1 expression, which is a protein that promotes autophagy and is a scaffold for the PI3K complex formation. Beclin-1 binds LC3I that is transformed to its membrane-bound form LC3-II, which then reacts with the ubiquitin-binding protein p62/sequestosome 1 [38,39,40]. Our data showed reduced Beclin-1 and LC3II protein expressions underlying an impaired autophagy during the disease.

An important role of autophagy is the elimination of aged or damaged organelles, such as mitochondria.

Mitophagy is the only degradative pathway able to eliminate damaged mitochondria [41]. Mitochondrial damage can arise from deposits of toxic species that may impair redox signaling or even inhibit mitochondrial function. Indeed, mitochondria are the key hub in the cell energy demand, apoptosis signaling and reactive species. Pink1 and Parkin play a key role in the elimination of damaged mitochondria. These two genes have been found often mutated in pathogenic conditions. Pink1 encrypts the PTEN-induced putative kinase with a mitochondrial targeting sequence, while Parkin is an E3 ubiquitin ligase [29]. Pink1 is involved in mitochondria homeostasis by acting as an initiator of mitophagy and a sensor of mitochondrial damage [42]. Parkin is an important mediator that selectively binds damaged mitochondria to induce them into degradation. In particular, mitochondrial health regulates Parkin intracellular location. Under normal conditions, this cytosolic protein translocates to depolarized mitochondria. Parkin translocation is Pink1 dependent. Pink1 levels in normal cells are low because they are cleaved and degraded in the mitochondrial inner membranes by PARL [43]. In the absence of PARL, Pink1 is not degraded, but stabilized on the outer mitochondrial membrane in order to recruit Parkin to impaired mitochondria [43]. Parkin translocation is further promoted by Bnip3 and Ambra1, which are autophagy-regulating proteins [44]. This mechanism allows Parkin and Pink1 to promote the turnover of damaged mitochondria. Once located in the organelle, Parkin promotes the colocalization of the autophagy marker LC3 [45]. Our data showed impaired mitophagy in endometriotic lesions. Rapamycin administration, by activating the mTOR pathway, restored the mitophagic mechanism in endometriotic lesions. In particular, we showed increased Bnip3, Ambra1 and Parkin expressions after rapamycin administration. Through these mitochondrial mediators, the Pink1–Parkin mechanism plays critical roles in preserving mitochondrial homeostasis through managing mitophagy and mitochondria-mediated apoptosis. Many papers showed that the apoptosis mechanism is impaired during endometriosis. Interestingly, autophagy and apoptosis are tightly regulated processes that can be triggered or inhibited by common signals [15,46,47,48]. Thus, while in many diseases the overactivation of both processes has been identified as detrimental, in similar pathology their activations showed a cytoprotective function [49,50,51]. Our study showed increased expression of the anti-apoptotic protein Bcl2 and reduced expression of pro-apoptotic protein Bax in the endometriotic lesions. Rapamycin administration, by activation the autophagy and mitophagy pathways, led to the activation of the apoptosis mechanism. Through the activation of mitophagy by rapamycin, cells activate pro-apoptotic Bcl-2 family proteins placed on the mitochondrial membrane. They, in turn, induced the release of cytochrome c from the mitochondrial intermembrane space into the cytosol, inducing apoptosis by activating caspases [52]. Mitophagy proteins Pink1 and Parkin, as well as Bcl-2 family proteins activation, would reduce endometriosis development.

Inhibition of endometriotic cell apoptosis enables lesion survival and is often accompanied with angiogenesis. Increasing evidence proposes that the induction of endometrial cell apoptosis may counteract angiogenesis [53]. Growth and development of the lesions required nutrients and oxygen supply; thus, during endometriosis, angiogenesis is significantly increased. In particular, endometriotic lesions showed increased VEGF and CD34 expressions. Strategies that counteract angiogenesis are important potential approaches to inhibit endometriosis. mTOR autophagy regulation by rapamycin leads to the direct inhibition of the proliferation of endothelial cells mediated by VEGF and CD34.

In conclusion, our data showed that the autophagy/mitophagy regulation of apoptosis/angiogenesis cross-talk is an important actor during endometriosis, and research in this direction will lead to novel insights into the etiology of this pathology.

The current research has some limitations. The employed endometriosis model was applied by transplanting normal rat uterine tissue into the abdominal cavity of another rat. It did not accurately represent the pathogenesis of human endometriosis. Indeed, the data were obtained with an artificial model (no spontaneous lesions, rats do not menstruate, no human lesion grafts), and their demonstration using rapamycin may not recapitulate the clinical/biological reality. However, rat models have a long history of being widely used in endometriosis research and have also been validated as a model that depicts the pathology dynamics. In future experiments it would be interesting to study the lesions for a longer period of time.

## 4. Materials and Methods

### 4.1. Animals

Female Sprague–Dawley rats (Envigo, Milan, Italy) were used in this research. The University of Messina Review Board for animal care (OPBA) approved the study. All animal experiments agreed with the new Italian regulations (D.Lgs 2014/26), EU regulations (EU Directive 2010/63) and the ARRIVE guidelines.

### 4.2. Experimental Protocol

Animals were randomly divided into two groups, donor or recipient, and endometriosis was established as already described [54]. To stimulate similar estrogen levels, donor rats were intraperitoneally injected with 10 IU pregnant mare serum gonadotropin to induce similar estrogen levels between various animals. The animals were euthanized 41 h later by CO_2_ asphyxia. A midline incision was performed, and the uterus was removed and washed in PBS. A longitudinal incision was made from one horn to the other. Tissue was then transferred to a 1.5 mL centrifuge tube containing fresh PBS and minced with scissors. Tissue from all donors was pooled, and the volume was adjusted to the equivalent of one uterus/500 µL of PBS. Recipient animals were injected intraperitoneally with the equivalent of tissue from one uterus in 500 µL of PBS along the midventral line. Endometriosis was allowed to develop for seven days. Seven days after the induction a group of animals was sacrificed to demonstrate the development of the lesions (Figure S1A–D).

### 4.3. Experimental Groups

Rats were randomized and assigned to the following groups:

(1) Vehicle group (*n* = 10): Rats were subjected to experimental endometriosis as described above, and vehicle (saline) was administered by gavage on the 7th day and for the next 7 days.
(2) Rapamycin group (*n* = 10): Rats were subjected to experimental endometriosis as described above, and rapamycin (0.5 mg/kg) was orally administered once per week.
(3) Sham group (*n* = 10): Rats were injected intraperitoneally with 500 µL of PBS without endometrial tissue, and vehicle (saline) was administered.

The dose of rapamycin was based on previous experiments [55].

The long elimination half-life of rapamycin necessitated a loading dose but allowed once-weekly administration without cell toxicity [56].

In order to evaluate the effect of rapamycin on the endometriotic lesions, rats were sacrificed at 14 days after endometriosis induction. Laparotomy was performed to collect the endometriotic implants and further processed for molecular analysis.

### 4.4. Histological Examination

For histopathological assessment, endometriosis lesions were fixed at room temperature in buffered formaldehyde solution, sections were stained with H&E and evaluated using a Leica DM6 microscope (Leica Microsystems SpA, Milan, Italy) equipped with a motorized stage and associated with Leica LAS X Navigator software (Leica Microsystems SpA, Milan, Italy) [57,58]. Histopathologic scores were performed by two investigators in a blind fashion. The whole image was quantified. The formula P (persistence of epithelial cells in the explants) × I (intensity of glands) was applied as described previously [49]: P: 3 = well-preserved epithelial layer; 2 = moderately preserved epithelium with leukocyte infiltrating; 1 = poorly preserved epithelium (occasional epithelial cells only); and 0 = no epithelium; I: from 0 (no glands) to 3 (abundant glands) [59]. Additionally, lesion volume was calculated according to the formula V = (length × width^2^) × 0.5.

### 4.5. Immunohistochemical Analysis

Immunohistochemical localization of VEGF and CD34 was performed as already described [16,60]. Lesion tissues were fixed in 10% (*w*/*v*) PBS-buffered formaldehyde and embedded in paraffin. Seven-micrometer sections were prepared from the samples. After deparaffinization, endogenous peroxidase was inactivated with 0.3% (*v*/*v*) H_2_O_2_ in 60% (*v*/*v*) methanol for 30 min. The sections were permeabilized with 0.1% (*w*/*v*) Triton X-100 in PBS for 20 min. Non-specific adsorption was decreased by incubating the section in 2% (*v*/*v*) normal goat serum in PBS for 20 min. Endogenous avidin or biotin binding sites were blocked by sequential incubation for 15 min with commercial avidin and biotin (Vector Laboratories, Burlingame, CA, USA), respectively. Subsequently, the sections were incubated overnight with primary antibodies: anti-VEGF antibody (1:250 in PBS *v*/*v*, Santa Cruz Biotechnology, Heidelberg, Germany, CGA7) and anti-CD34 antibody (1:250 in PBS *v*/*v*, Santa Cruz Biotechnology, Heidelberg, Germany, sc-74499). All sections were washed with PBS and incubated with peroxidase-conjugated bovine anti-mouse IgG, secondary antibody (1:2000 Jackson Immuno Research, West Grove, PA, USA). Specific labeling was provided with a biotin-conjugated goat anti-mouse IgG and avidin-biotin peroxidase complex (Vector Laboratories, Burlingame, CA, USA) [61]. Liver tissue was used as VEGF-positive control (Figure S1E), and kidney tissue was used as a positive control for CD34 expression (Figure S1H), whereas negative controls were achieved by substituting the primary antibody with normal goat serum (Figure S1F,I) or PBS (Figure S1G,J) [62,63] (Figure S1). Stained sections were observed using a Leica DM6 microscope following a typical procedure [64]. The histogram profile is related to the positive pixel intensity value obtained [65]. The figures shown are representative of at least three experiments performed on different days and on 5 tissue sections collected from 5 animals in each group.

### 4.6. Western Blot Analysis

Western blots were performed as already described [66,67]. Specific primary antibody anti-mTOR (Cell Signaling, Danvers, MA, USA, 2972), anti-Beclin (Santa Cruz Biotechnology, Heidelberg, Germany, sc-48381), anti-LC3 II (Sigma Aldrich, Munich, Germany, ABC232), anti-p-AKT (Santa Cruz Biotechnology, Heidelberg, Germany, sc-293125), anti-Bnip3 (Abcam, Cambridge, MA, USA, ab104343), anti-Ambra1 (Abcam, Ab69501), anti-Parkin (Santa Cruz Biotecnology, Heidelberg, Germany, sc-32282), anti-Bcl-2 (Santa Cruz Biotechnology, Heidelberg, Germany, sc-7382), anti-Bax (Santa Cruz Biotecnology, Heidelberg, Germany, sc-7480), or anti-cleaved caspase 3 (Santa Cruz Biotechnology, Heidelberg, Germany, sc-271028) was mixed in 5% *w*/*v* nonfat dried milk solution and was incubated at 4 °C, overnight. Blots were incubated with peroxidase-conjugated bovine antimouse IgG secondary antibody or peroxidase-conjugated goat antirabbit IgG (Jackson Immuno Research) for 1 h at room temperature [68,69]. To verify the equal amounts of protein, membranes were also incubated with the antibody against β-actin (Santa Cruz Biotechnology). Signals were detected with enhanced chemiluminescence detection system reagent (Super-Signal West Pico Chemiluminescent Substrate, Pierce, Monza, Italy) [70,71]. The relative expression of the protein bands was quantified by densitometry with Bio-Rad ChemiDoc XRS software. Images of blot signals were imported to analysis software (Image Quant TL, Amersham Biosciences, Freiburg, Germany, v2003) [72]. The Western blot analyses are representative of 3 different gels made by dividing the number of samples obtained from 5 animals for each experimental group in different days.

### 4.7. Statistical Evaluation

All values are expressed as mean ± standard error of the mean (SEM) of *n* observations. For in vivo studies, *n* represents the number of animals used. The results were analyzed by t-test, and the Kolmogorov–Smirnov test was applied to analyze the normal distribution of the data (Prism 8 for macOS version 8.2.1 (279)). A *p* value of less than 0.05 was considered significant. * *p* < 0.05 vs. vehicle, ** *p* < 0.01 vs. vehicle, *** *p* < 0.001 vs. vehicle.

## Data Availability

The data presented in this study are available on request from the corresponding author.

## Supplementary Materials

The following are available online at https://www.mdpi.com/article/10.3390/ijms22105074/s1.
