# Supplementary figures and images for: Autophagy and Mitophagy Promotion in a Rat Model of Endometriosis
Source: Int J Mol Sci. 2021 May 11;22(10):5074. doi: 10.3390/ijms22105074 (PMC8150724; doi:10.3390/ijms22105074)

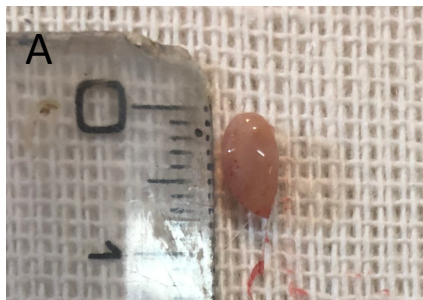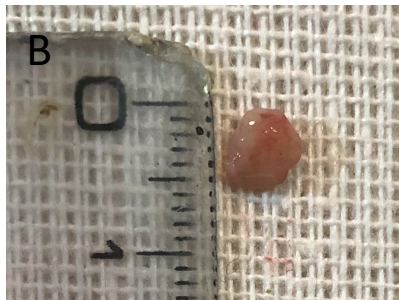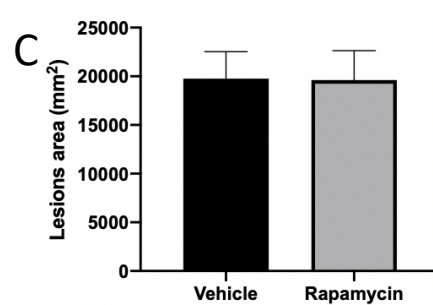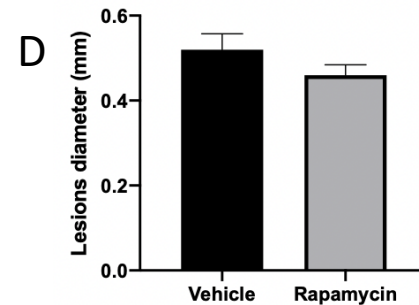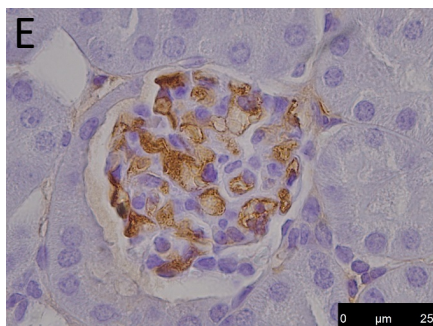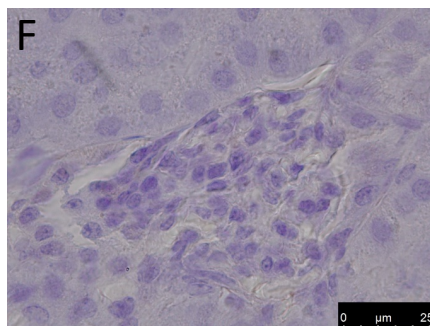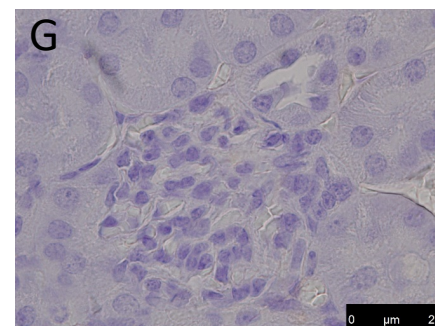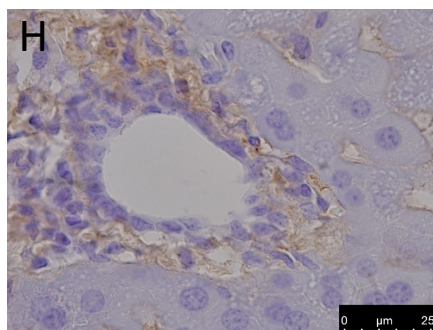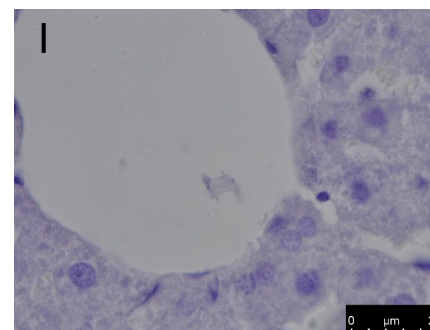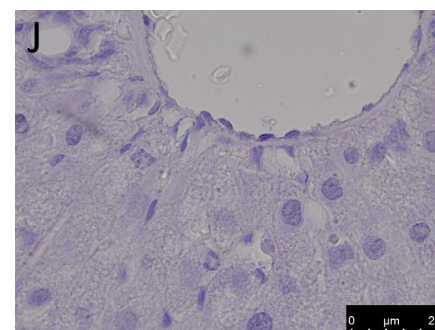

Supplement: Supplementary file 1 [file ijms-22-05074-s001.zip › ijms-1213357-supplementary.pdf]
